# Supplementary material for: Altered DNA methylation profiles in blood from patients with sporadic Creutzfeldt–Jakob disease
Source: Acta Neuropathol. 2020 Sep 12;140(6):863–79. doi: 10.1007/s00401-020-02224-9 (PMC7666287; doi:10.1007/s00401-020-02224-9)
Supplement: Supplementary file 1 — Additional file1 (PDF 26864 kb) [file 401_2020_2224_MOESM1_ESM.pdf]

Altered DNA methylation profiles in blood from patients with sporadic Creutzfeldt-Jakob disease

Luke Dabin[1¥], Fernando Guntoro[1¥], Tracy Campbell[1], Tony Bélicard[2], Adam R. Smith[3], Rebecca G. Smith [3], Rachel Raybould[4], Jonathan M. Schott[5], Katie Lunnon[3], Peter Sarkies[2], John Collinge[1], Simon Mead[1] and Emmanuelle Viré[1].

[¥]Denotes equal contribution

Corresponding author: s.mead@ucl.ac.uk

1. MRC Prion Unit at UCL, UCL Institute of Prion Diseases, Courtauld Building, London, W1W 7FF, UK.
2. MRC London Institute of Medical Sciences, London W12 0NN, UK; Institute of Clinical Sciences, Imperial College London, London W12 0NN, UK.
3. College of Medicine and Health, University of Exeter Medical School, Exeter University, RILD Building Level 4, Royal Devon and Exeter Hospital, Barrack Rd, Exeter, EX2 5DW, UK.
4. Institute of Psychological Medicine and Clinical Neurosciences, Cardiff University, Cardiff, UK
5. Dementia Research Centre, UCL Institute of Neurology, University College London, London, UK.

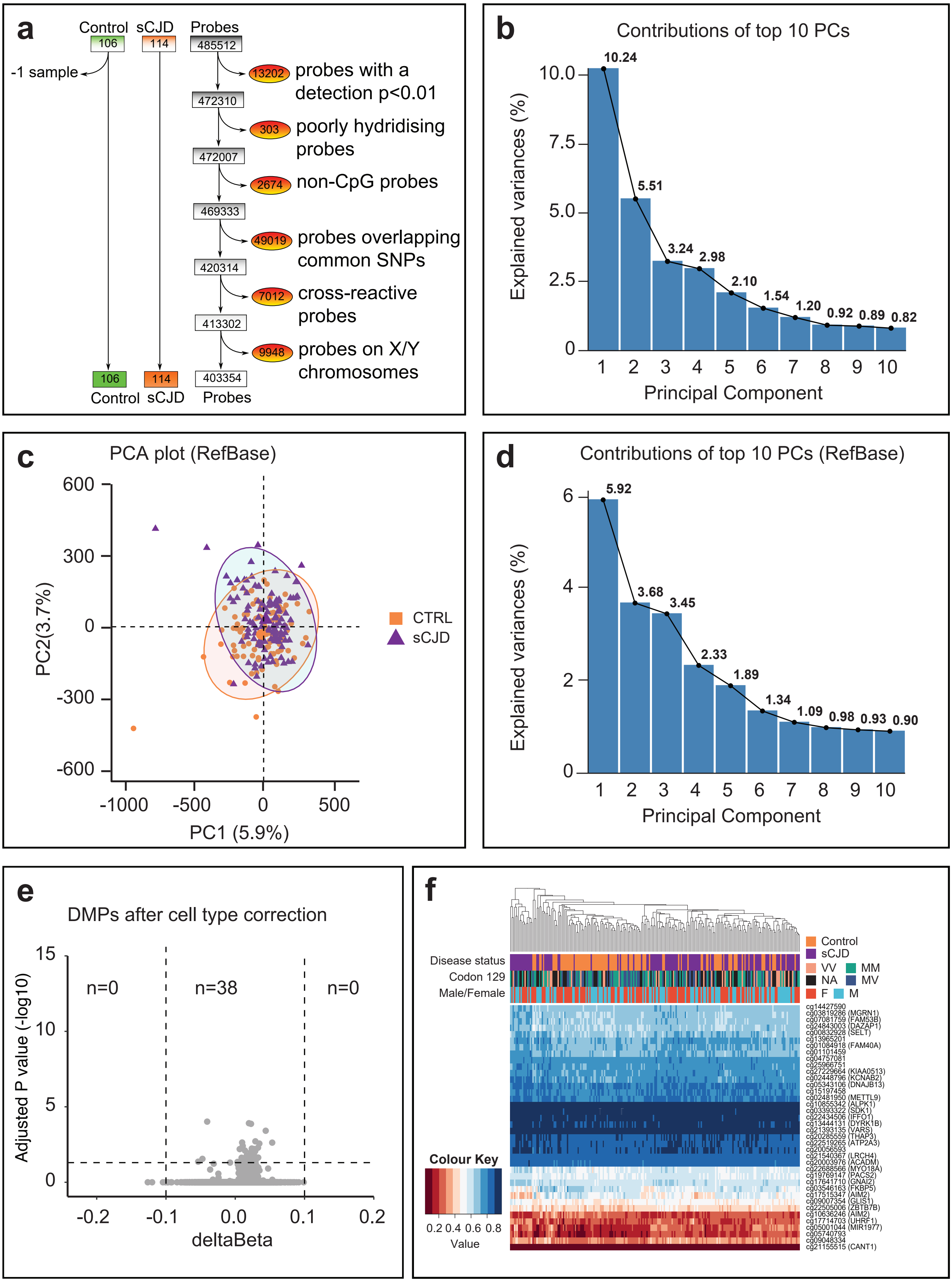
a
Control
sCJD
Probes
106
114
485512
-1 sample
13202
probes with a detection p<0.01
472310
303
poorly hydridising probes
472007
2674
non-CpG probes
469333
49019
probes overlapping common SNPs
420314
7012
cross-reactive probes
413302
9948
probes on X/Y chromosomes
106
114
403354
Control
sCJD
Probes
b
Contributions of top 10 PCs
Explained variances (%)
Principal Component
10.24
5.51
3.24
2.98
2.10
1.54
1.20
0.92
0.89
0.82
c
PCA plot (RefBase)
PC2(3.7%)
PC1 (5.9%)
CTRL
sCJD
d
Contributions of top 10 PCs (RefBase)
Explained variances (%)
Principal Component
5.92
3.68
3.45
2.33
1.89
1.34
1.09
0.98
0.93
0.90
e
DMPs after cell type correction
Adjusted P value (-log10)
deltaBeta
n=0
n=38
n=0
f
Disease status
Codon 129
Male/Female
Control
sCJD
VV
MM
NA
MV
F
M
Colour Key
Value
cg14427590
cg03819286 (MGRN1)
cg07081759 (FAM53B)
cg24843003 (DAZAP1)
cg00832928 (SELT)
cg13965201
cg01084918 (FAM40A)
cg01101459
cg04757081
cg25966751
cg27229664 (KIAA0513)
cg02448796 (KCNAB2)
cg05343106 (DNAJB13)
cg15197458
cg02481950 (METTL9)
cg10855342 (ALPK1)
cg03393322 (SDK1)
cg22434506 (IFFO1)
cg13444131 (DYRK1B)
cg21393135 (VARS)
cg20285559 (THAP3)
cg22519265 (ATP2A3)
cg20056593
cg21540367 (LRCH4)
cg20003976 (ACADM)
cg22688566 (MYO18A)
cg19769147 (PACS2)
cg17641710 (GNAI2)
cg03546163 (FKBP5)
cg17515347 (AIM2)
cg09007354 (GLIS1)
cg22505006 (ZBTB7B)
cg10636246 (AIM2)
cg17714703 (UHRF1)
cg05001044 (MIR1977)
cg05740793
cg09048334
cg21155515 (CANT1)

Supplementary Figure 1: Genome-wide methylation profiling in sporadic CJD blood.

(**a**) Graphical representation of quality control steps; 82158 probes and one sample were excluded after performing the champ.load() function. (**b**) Scree plot showing the percentage of total variance in the methylation profiles as represented by the top 10 most contributing PCs (cell type corrected) before cell type correction. (**c**) Principal component analysis (PCA) showing the first (PC1) and second (PC2) principal components (PC) for 219 DNA methylation profiles after cell type correction with Housemann algorithm. Control samples (orange) and sCJD cases (purple). (**d**) Scree plot showing the percentage of total variance in the methylation profiles as represented by the top 10 most contributing PCs (cell type corrected) after cell type correction with Housemann algorithm. (**e**) Volcano plot showing differences in methylation levels between sCJD and control after cell type correction. (**f**) Dendrogram showing hierarchical clustering of the 38 significant DMPs. Hierarchical clustering analysis was performed using the average clustering method based on Euclidean distance. Disease status: orange (control), purple (sCJD); codon 129 status: MM (green), MV (dark blue), VV (pink), unknown (black); sex: male (blue), female (red).

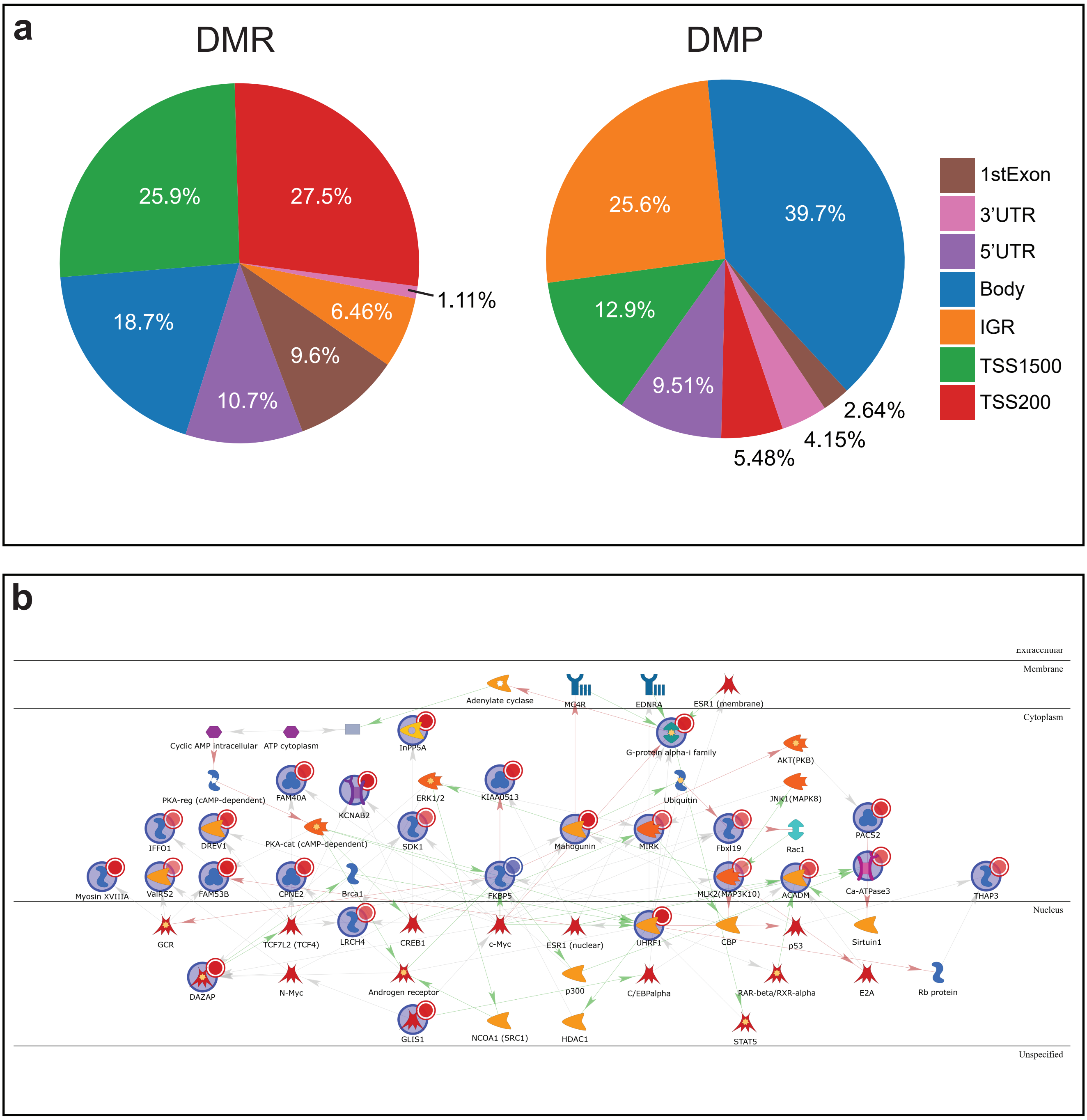

Supplementary Figure 2: Characterization of DMPs and DMRs.

(**a**) Distribution of CpGs in the38 DMPs (right) and 67 DMRs (left). The segments are ordered according to the size of proportions. TSS 1500: located 1500 nucleotides upstream transcriptional start site; TSS 200: located 1500 nucleotides upstream transcriptional start site; Body: body of the gene; IGR: intergenic regions; 5'UTR and 3'UTR are respectively 5' and 3' untranslated regions. (**b**) Pathways affected by changes in DNA methylation in sCJD. Molecules are stratified by subcellular localisation. Green arrows represent activation, red represent inhibition, and grey arrows represent unspecific interaction. Molecules encircled in blue are from the methylation dataset. Upper-right coloured circles represent effect direction, with red circles indicating hypermethylation and blue circles representing hypomethylation in sCJD. Proteins are represented by blue molecular shapes, enzymes are represented by yellow and orange shapes, and transcription factors are represented by red shapes.

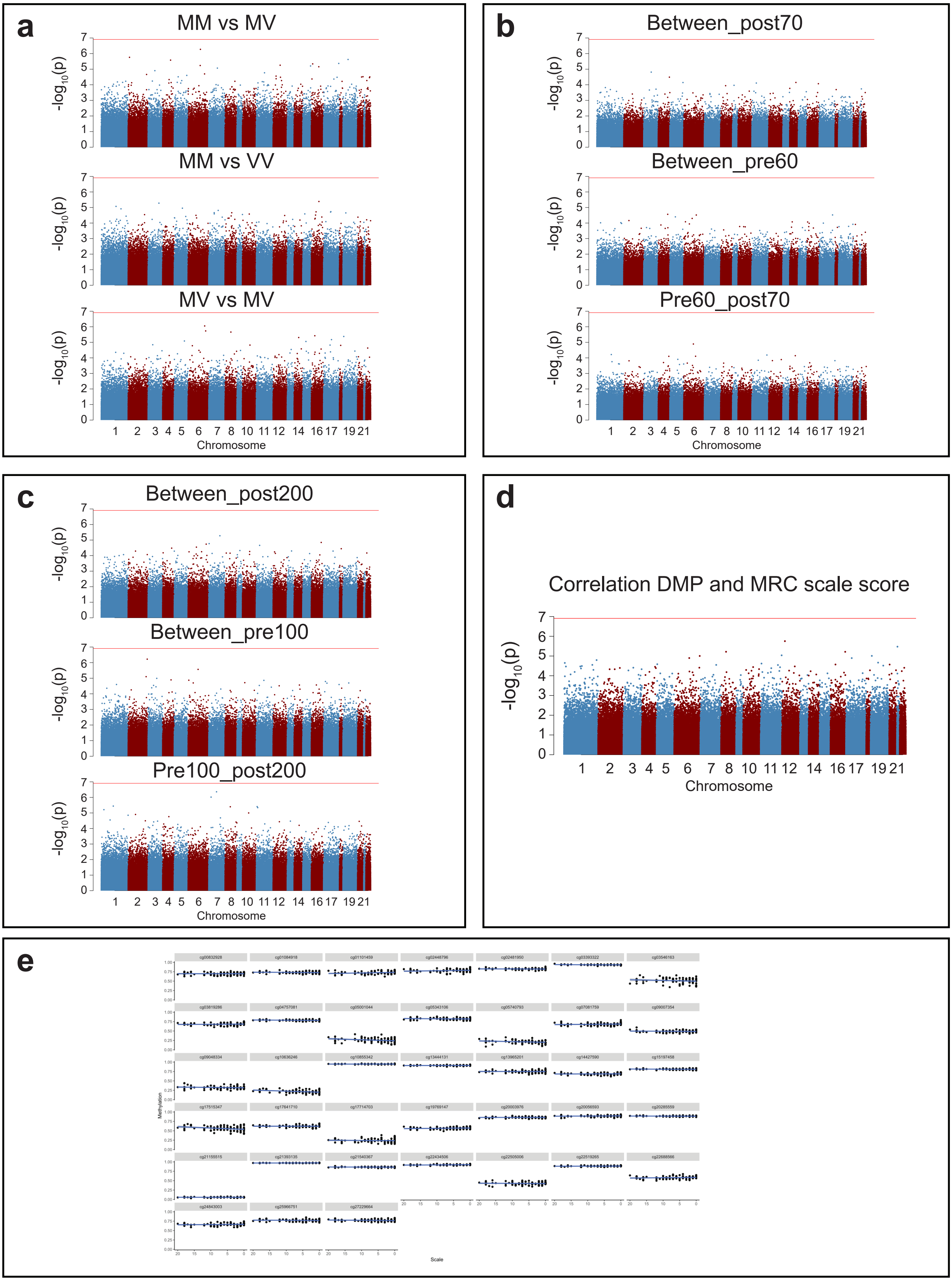
a
MM vs MV
MM vs VV
MV vs MV
b
Between_post70
Between_pre60
Pre60_post70
c
Between_post200
Between_pre100
Pre100_post200
d
Correlation DMP and MRC scale score
-log10(p)
Chromosome
1 2 3 4 5 6 7 8 10 11 12 14 16 17 19 21
e
Methylation
Scale

Supplementary Figure 3: Correlation between 38 DMPs and disease-modifying features. (**a**) Manhattan plot of probes associated with *PRNP* codon 129 polymorphism. Red line indicates significance threshold (Bonferroni-adjusted = $1.24x10^{-7}$). X-axis represents ranked chromosomes, Y-axis represents–log10 (p-value). (**b**) Manhattan plot of probes associated with age at onset. Cases are binned in three groups: onset before 60 years old; onset between 60 and 70 years old; onset after 70 years old. Red line indicates significance threshold (Bonferroni-adjusted = $1.24x10^{-7}$). X-axis represents ranked chromosomes, Y-axis represents–log10 (p-value). (**c**) Manhattan plot of probes associated with disease duration. Cases are binned in three groups: less than 100 days, longer than 200 days, or between 100 and 200 days. Red line indicates significance threshold (Bonferroni-adjusted = $1.24x10^{-7}$). X-axis represents ranked chromosomes, Y-axis represents–log10 (p-value). (**d**) Manhattan plot of probes associated with patient's score measured at the time each sample was collected. Red line indicates significance threshold (Bonferroni-adjusted = $1.24x10^{-7}$). X-axis represents ranked chromosomes, Y-axis represents–log10 (p-value). (**e**) Correlation between the patient's score measured at the time each sample was collected with the methylation values at each DMP.

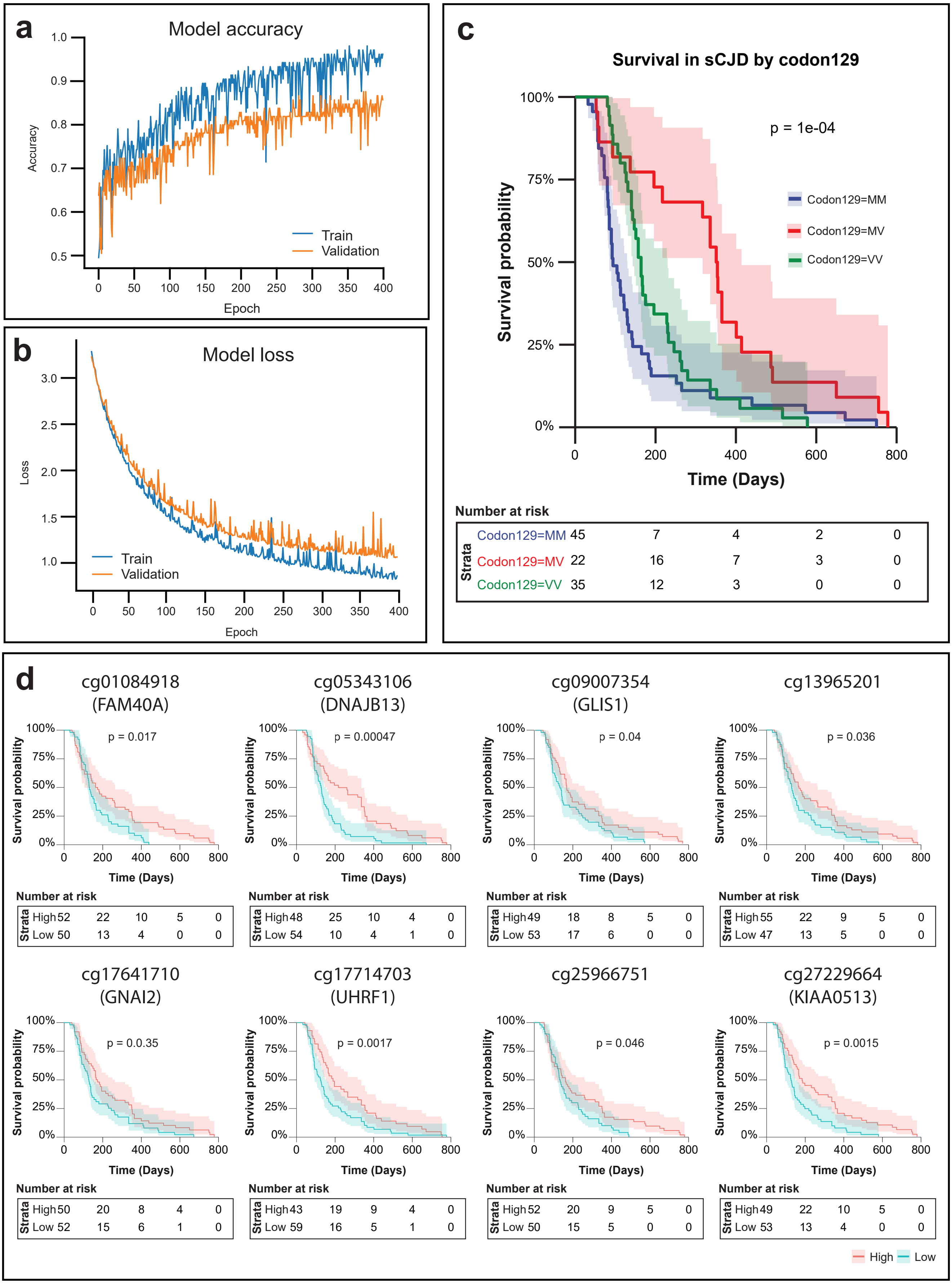
a
Model accuracy
Accuracy
Epoch
Train
Validation
b
Model loss
Loss
Epoch
Train
Validation
c
Survival in sCJD by codon129
p = 1e-04
Survival probability
Time (Days)
Codon129=MM
Codon129=MV
Codon129=VV
Number at risk
Strata
Codon129=MM 45 7 4 2 0
Codon129=MV 22 16 7 3 0
Codon129=VV 35 12 3 0 0
d
cg01084918 (FAM40A)
p = 0.017
High 52 22 10 5 0
Low 50 13 4 0 0
cg05343106 (DNAJB13)
p = 0.00047
High 48 25 10 4 0
Low 54 10 4 1 0
cg09007354 (GLIS1)
p = 0.04
High 49 18 8 5 0
Low 53 17 6 0 0
cg13965201
p = 0.036
High 55 22 9 5 0
Low 47 13 5 0 0
cg17641710 (GNAI2)
p = 0.0.35
High 50 20 8 4 0
Low 52 15 6 1 0
cg17714703 (UHRF1)
p = 0.0017
High 43 19 9 4 0
Low 59 16 5 1 0
cg25966751
p = 0.046
High 52 20 9 5 0
Low 50 15 5 0 0
cg27229664 (KIAA0513)
p = 0.0015
High 49 22 10 5 0
Low 53 13 4 0 0
Survival probability
Time (Days)
Number at risk
Strata
High
Low

Supplementary Figure 4: DNA methylation array profiles to refine sCJD diagnosis and disease duration.

Performance of DNA methylation-based neural network classifier in sCJD. (**a**) Model accuracy during training process over 400 epochs. (**b**) Model loss during the training process. The loss is defined as binary cross-entropy. (**c**) Kaplan-Meier survival analysis. sCJD patients were divided into 3 groups based on the genotype at PRNP codon 129 (MM, VV or MV). (**d**) Survival analysis for 8 sites. Patients were divided into high (above median; red) and low (below media; green) DNA methylation values at each DMP. *p*-values were calculated using the log-rank test.
